# Corrosion Electrochemistry and Protection of Metallic Materials

**DOI:** 10.3390/ma19214718

**Published:** 2026-11-06

**Authors:** Citlalli Gaona-Tiburcio, Ricardo Galvan-Martínez, Facundo Almeraya-Calderón

**Affiliations:** 1 Universidad Autónoma de Nuevo León, FIME, Centro de Investigación e Innovación en Ingeniería Aeronáutica (CIIIA), San Nicolás de los Garza 66455, Mexico; citlalli.gaonatbr@uanl.edu.mx; 2 Instituto de Ingeniería, Universidad Veracruzana, Calz Juan Pablo II S/N, Fracc. Costa Verde, Veracruz 94294, Mexico; rigalvan@uv.mx

## 1. Introduction and Scope

Metallic alloys are widely used in the automotive, aerospace, aeronautical, chemical, and marine industries, where components must meet stringent performance and durability requirements. These applications require materials with adequate mechanical strength, fatigue resistance, and, particularly, corrosion resistance. Corrosion-related degradation remains a major industrial challenge with economic, safety, and logistical consequences. Mechanical strength alone cannot ensure the durability of metallic alloys exposed to aggressive environments. Improving their performance requires optimization of alloy composition and metallurgical processing in addition to effective corrosion-control strategies, including development of protective coatings. Such approaches can extend metallic components’ service lives.

This Special Issue, “Corrosion Electrochemistry and Protection of Metallic Materials”, presents recent research on electrochemical corrosion science, engineering, and technology. It brings together multidisciplinary investigations of metallic materials, emphasizing electrochemical characterization techniques, corrosion mechanisms, and methods for corrosion prevention and protection.

## 2. Contributions

This Special Issue brings together original research contributions addressing electrochemical corrosion processes and strategies for protecting metallic materials. It comprises ten research articles covering a range of alloys, exposure environments, characterization methods, and protective approaches.

Liu, S., et al. (Contribution 1) systematically investigated the corrosion and stress corrosion cracking (SCC) behavior of L415 pipeline steel in a simulated soil environment representative of the Gannan region in Southern China. Corrosion kinetics were strongly influenced by environmental chemistry: the corrosion rate increased from 0.0505 to 0.1949 mm/year under the combined effects of low pH and high sulfate concentrations [1].

Martinez-Ramos, C., et al. (Contribution 2) investigated the use of hidden Markov models (HMMs) to classify and reconstruct corrosion mechanisms in the aerospace-grade aluminum alloy AA2055 using electrochemical noise (EN) signals. Their findings demonstrate that meaningful signal reconstruction was possible despite variations in signal quality, particularly when datasets contained distinct transient features [2].

Bao, M., et al. (Contribution 3) investigated the corrosion behavior of L245 pipeline steel in chloride-containing solutions with varying thiosulfate (S_2_O_3_^2−^) concentrations. Weight-loss measurements at different exposure times showed that thiosulfate promoted localized corrosion and that the corrosion behavior depended on its concentration in the chloride-containing solution [3].

Li, B., et al. (Contribution 4) examined Cu–Sn alloys in 3.5 wt.% NaCl and simulated archaeological soil solutions using electrochemical methods, X-ray diffraction (XRD), and X-ray photoelectron spectroscopy (XPS). Chloride ions played a key role in the formation of detrimental corrosion products, including Cu_2_(OH)_3_Cl. Chloride-containing corrosion products also increased the wettability of the rust layers, facilitating penetration by corrosive species and promoting further degradation [4].

Bonfil, D., et al. (Contribution 5) investigated the effect of chlorides on the corrosion behavior of AISI 304 stainless steel and A36 carbon steel in an extract of a hybrid pumice–Portland cement system containing 5 g/L of NaCl, which was used to simulate a chloride-contaminated concrete pore environment. The extract pH decreased from 12.99 to 9.5 after 14 days, indicating an increased corrosion risk for A36 steel, whose open-circuit potential (OCP) was approximately −363 mV. In contrast, stainless steel 304 exhibited passivation behavior, with its OCP shifting to approximately +72 mV [5].

Alontseva, D., et al. (Contribution 6) evaluated pure Ti and Ti_6_Al_4_V coatings formed in situ on 316L stainless steel via hot pressing. Corrosion and wear behavior were examined in Hanks’ solution and physiological saline (0.9% NaCl). Both coatings improved corrosion resistance relative to the uncoated 316L substrate, while wet wear tests showed lower friction coefficients in physiological saline solution than in Hanks’ solution [6].

Cieślik, M., et al. (Contribution 7) investigated the influence of mechanical stress on pitting corrosion initiation and the protective properties of passive films on AISI 304L stainless steel. In 3.5% NaCl solution, no initiation of pitting corrosion was observed in specimens loaded within either the elastic or plastic range under the conditions examined [7].

Alvarez-Malpica, J., et al. (Contribution 8) synthesized a long-chain aliphatic amine compound and evaluated its ability to inhibit steel corrosion in a sour saline environment.

At an inhibitor concentration of 20 ppm, no significant change in inhibition efficiency was observed. At 100 ppm, however, the inhibition efficiency exceeded 95% [8].

Stejskal, J., et al. (Contribution 9) investigated iron microparticles coated in situ with polypyrrole during the chemical oxidation of pyrrole with ammonium peroxydisulfate in an aqueous medium. Scanning electron microscopy revealed the coating’s morphology, while Fourier-transform infrared (FTIR) and Raman spectroscopy provided evidence of its molecular structure and coverage. The study also describes morphological differences between microscopic and macroscopic polypyrrole structures [9].

Cabral-Miramontes, J., et al. (Contribution 10) investigated hard-anodized coatings on aluminum alloys 2024-T3 (Al–Cu), 6061-T6 (Al–Mg–Si), and 7075-T6 (Al–Zn) produced using citric- and sulfuric-acid baths at different concentrations. Among the alloys studied, 6061-T6 exhibited the greatest coating thickness and hardness [10].
